# Design, development and characterization of resistive arm based planar and conformal metasurfaces for RCS reduction

**DOI:** 10.1038/s41598-022-19075-x

**Published:** 2022-09-02

**Authors:** Priyanka Tiwari, Surya Kumar Pathak, Varsha Siju

**Affiliations:** 1 Institute for Plasma Research, Bhat, Gandhinagar, Gujarat 382428 India; 2 Homi Bhabha National Institute, Training School Complex, Anushaktinagar, Mumbai, 400094 India

**Keywords:** Electrical and electronic engineering, Aerospace engineering

## Abstract

This paper reports the design, development and characterization of a broadband, polarization insensitive metasurface absorber in planar, cylindrically bent and 90° dihedral surface geometry. Four metallic patches loaded with eight lumped resistors are used, which has been optimized numerically using CST MICROWAVE STUDIO, as a unit cell of developed metasurface absorber, to achieve 20 dB reflection reduction for 51.21% fractional bandwidth (13.42–22.66 GHz) under normal incidence with 0.12 ${\lambda }_{L}$ thickness (where ${\lambda }_{L}$ corresponds to lower operating frequency). The numerical findings are also verified analytically using equivalent circuit analysis, which exhibits very good agreement. Polarization-insensitive characteristics are achieved using fourfold rotation symmetry of the designed structure. The fabricated prototype of the designed absorber is experimentally characterized, using free space measurement method and AB*mm* vector network analyzer (VNA) system, and fairly good agreement with numerical-analytical findings are reported. The major novelty of this study is the design and development of a broadband (13.42–22.66 GHz), polarization insensitive metasurface absorber that provides 20 dB reflection reduction numerically as well as experimentally in the whole band, which to the author’s knowledge has not been observed till now. Also, keeping in mind the radar stealth applications, first time we have demonstrated both numerically and experimentally, different geometrical shapes of conformal metasurfaces that can be practically used in actual scenario.

## Introduction

The radar cross section (RCS) reduction is the most efficient method to escape from enemy radar and thus helps aircraft in surveillance. Many techniques have been developed in the past that focus on the RCS reduction of potential target like purpose shaping, active cancellation, radar absorbing material (RAM)^1^ etc. The λ/4 Salisbury screen^2^ is one of the simplest and oldest resonant type RAM, which exhibits very narrow band absorption characteristic. Jaumann absorber^3^, extension of the Salisbury screen, enhances absorption bandwidth but at the same time increases total weight and thickness. Development of metasurface based absorber^4–6^, overcomes the drawback of conventional microwave absorber related to larger thickness, bulky size, heavyweight and larger surface area which was an earlier constraint for space-related application. In past years, many absorbers have been proposed to increase the absorption bandwidth^7–23^. A multi-resonating absorber with improved bandwidth has been presented in^7–10^. Although these methods are comparatively easier to implement but at the expense of increased unit cell size and thickness of absorber which makes the device bulkier and thus limit its use in practical applications. To further improve absorption bandwidth, a conductive pattern with lossy element periodically printed on top of dielectric substrate backed by metal, also known as circuit analogue (CA) absorber, has been developed^11^. Lossy element in CA absorber can be implemented by using a lumped resistor with a specific value. In previous studies^12–19^, CA absorber based on lumped resistor shows 10 dB reflection reduction, while^20^ shows 20 dB reflection reduction with fractional bandwidth (FBW) 40%. Although^19^ proposed 83.4% FBW for 20 dB reflection reduction but experimental characterization is reported only for 10 dB reduction. The use of resistive ink patterns is another method to implement lossy element^21–23^. CA absorber based on resistive ink exhibits 10 dB reflection reduction in^21^ and 15 dB reflection reduction in^22^. Also, in^23^, 20 dB reflection reduction is proposed, but experimental validation has been done only for 10 dB reflection reduction. Another method based on artificial magnetic conductor (AMC) to reduce RCS has been reported in^24–26^. In^24^ and^25^, 63% and 91%, respectively, 10 dB RCS reduction bandwidth is reported. Both these checkerboard AMC design,^24^ and^25^ are polarization sensitive. In^26^, a polarization convertor metasurface (PCM) has been used to reduce RCS of planar surface. Recently, RCS reduction capability of a resistive-ink based metasurface absorber has been presented in^27^, but experimental validation has been reported only for planar surface. In^28,29^, diffusive scattering based metasurface has been used to reduce RCS. A different method to develop invisibility cloaks for stealth application is recently proposed in^30^.

In radar stealth applications, the value of RCS determines the maximum detection range of a potential target^1^. In other words, if the detection range for 0 dB RCS reduction value is 100 miles (arbitrary), then it will reduce to 56 miles for a 10 dB RCS reduction value and 32 miles for a 20 dB RCS reduction value^1^. Therefore, if the RCS value of the target reduces, its maximum detection range also reduces, which results in reduction in the reaction time assigned to the enemy’s radar system^1^. In past years, most of the reported designs present 10 dB reflection reduction value which is not sufficient for highly sensitive defence application. Only a few reported studies proposed a computational design of 20 dB reflection reduction value, however, their experimental characterization has not been reported. In addition, the potential target in defence application have planar as well as non-planar surfaces, therefore it is necessary to study the performance of the proposed metasurface absorber on conformal surfaces, as well. In view of this, 20 dB reflection reduction value with broad fractional bandwidth as well as the development of different geometrical shapes of metasurfaces eventually become important criteria to qualify the proposed metasurface absorber as a standard absorbing device in radar stealth applications. All the previously reported works have limitation in terms of reflection reduction value, fractional bandwidth and different geometrical shapes of conformal metasurface. To address these issues as well as to avoid the detection range of highly sensitive Radars, first time we have designed, developed and characterized a broadband, polarization-insensitive metasurface absorber (MA) which exhibits 20 dB reflection reduction with 0.12 ${\uplambda }_{\mathrm{L}}$ thickness for 51.21% FBW (13.42–22.66 GHz). Additionally, RCS reduction capabilities of different geometrical shapes of conformal metasurface (planar, cylindrically bent and 90° dihedral surface) have also been characterized numerically and experimentally.

## Simulation results

### Design and equivalent circuit modeling

**Figure 1:**
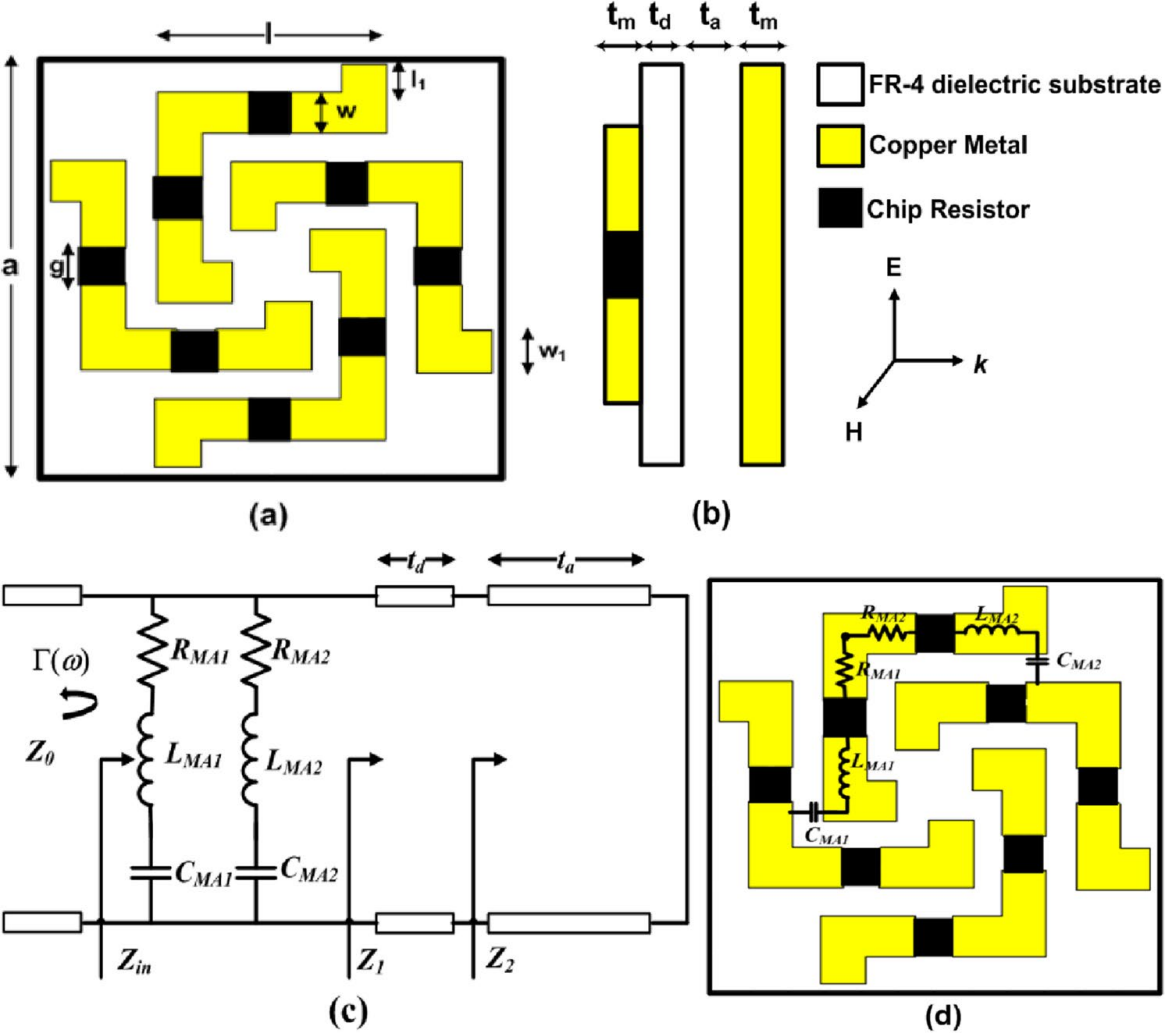
Unit cell geometry of the proposed absorber: (**a**) top view and (**b**) side view and (**c**) equivalent circuit model of the proposed metasurface absorber and (**d**) RLC modelling of top resistive layer.

Top view and side view of the unit cell geometry of the proposed metasurface absorber along with the direction of electric field, magnetic field and propagation of incident electromagnetic (EM) wave are shown in Fig. 1a,b, respectively. Design consist of four metallic patches loaded with eight surface mounted chip resistors on top of FR-4 dielectric substrate (*ε*_*r*_ = 4.3 and *tanδ* = 0.025) having thickness (t_d_) of 0.2 mm, which is separated by metallic ground plate using an air spacer with thickness (t_a_) of 2.6 mm. Copper with thickness (t_m_) of 0.035 mm and conductivity of 5.8 × 10^7^ S/m has been used for both top metallic patch and bottom ground plate. The optimized parameter of the unit cell are a = 10.93 mm, l = 6.04 mm, w = 1.17 mm, l_1_ = 0.4 mm, w_1_ = 0.5 mm, g = 2 mm and R = 150 Ω (value of chip resistor).

For normal incidence, absorptivity/absorption coefficient of metasurface absorber, $\mathrm{A}\left(\omega \right)$ is determined using Eq. (1)

1 $$\mathrm{A}\left(\omega \right)=1 -{\left|\Gamma \left(\omega \right)\right|}^{2}-{\left|\mathrm{T}\left(\omega \right)\right|}^{2}$$

where ${\left|\Gamma (\omega )\right|}^{2}$ is reflected power and Γ(*ω*) is reflection coefficient while ${\left|\mathrm{T}(\omega )\right|}^{2}$ is transmitted power and T(*ω*) is transmission coefficient, all of which depend on incident EM wave frequency $\omega$. A perfect metasurface based absorber can be realized by maximizing the absorption coefficient. This can be done by minimizing the reflection and transmission coefficient. The reflection coefficient, Γ(*ω*), can be suppressed to zero by optimizing the geometrical dimension of unit cell in such a way that impedance of absorber matched with impedance of free space Z_0_. By using metallic ground plate, with thickness much greater than skin depth, transmission from the structure T($\omega$) becomes zero and thus absorptivity/absorption coefficient of metasurface absorber $\mathrm{A}\left(\omega \right)$ reduces to

2 $$A\left(\omega \right)=1 -{\left|\Gamma \left(\omega \right)\right|}^{2}$$

The equivalent circuit model of the proposed absorbing structure is shown in Fig. 1c, where top metallic layer loaded with chip resistors is represented as two RLC series circuits connected in parallel. Here ${R}_{MAi}$, ${L}_{MAi}$ and ${C}_{MAi}$ represent the equivalent losses within the metallic patch loaded with chip resistor, inductance created due to the current path in the metallic patch, and the capacitance created due to the gap between two neighboring elements of unit cells, respectively, where i = 1 is for vertical resistive arm while i = 2 is for horizontal resistive arm as shown in Fig. 1d. Impedance of this layer (${Z}_{MA}$) can be written as

3 $$\begin{gathered} Z_{MA} = \left( { R_{MA1} + j\omega L_{MA1} - \frac{j}{{\omega C_{MA1} }}} \right) || \left( {R_{MA2} + j\omega L_{MA2} - \frac{j}{{\omega C_{MA2} }} } \right) \hfill \\ = \frac{{\left( { R_{MA1} + j\omega L_{MA1} - \frac{j}{{\omega C_{MA1} }}} \right) \left( {R_{MA2} + j\omega L_{MA2} - \frac{j}{{\omega C_{MA2} }}} \right) }}{{\left( { R_{MA1} + j\omega L_{MA1} - \frac{j}{{\omega C_{MA1} }}} \right) + \left( {R_{MA2} + j\omega L_{MA2} - \frac{j}{{\omega C_{MA2} }}} \right) }} \hfill \\ \end{gathered}$$

Air spacer with metallic ground plate is represented as short circuited transmission line of length *t*_*a*_ and input impedance seen from top of this air spacer (${Z}_{2}$) is defined as

4 $$Z_{2} = j\left( {\frac{{Z_{0} }}{{\sqrt {\varepsilon_{r2} } }}} \right){\text{tan}}(\beta_{2} t_{a} ) = j\left( {\frac{{Z_{0} }}{{\sqrt {\varepsilon_{r2} } }}} \right){\text{tan}}\left( {\left( {\omega \sqrt {\varepsilon_{r2} } } \right)t_{a} /c} \right)$$

where,${Z}_{0}$ is characteristic impedance of the free space, ${\beta }_{2}=(\omega \sqrt{{\varepsilon }_{r2}})/c$ is phase constant, ${\varepsilon }_{r2}$ is complex dielectric constant, ${t}_{a}$ is thickness of air spacer with metal ground plate and c is the velocity of light in free space. FR-4 dielectric substrate represented by transmission line having length *t*_*d*_ and input impedance seen from top of it (${Z}_{1}$) can be written as

5 $${Z}_{1}=\frac{{Z}_{0}}{\sqrt{{\varepsilon }_{r1}}}\frac{{Z}_{2}+j\frac{{Z}_{0}}{\sqrt{{\varepsilon }_{r1}}}\mathrm{tan}({\beta }_{1}{t}_{d})}{\frac{{Z}_{0}}{\sqrt{{\varepsilon }_{r1}}}+j{Z}_{2}\mathrm{tan}({\beta }_{1}{t}_{d})}= \frac{{Z}_{0}}{\sqrt{{\varepsilon }_{r1}}}\frac{{Z}_{2}+j\frac{{Z}_{0}}{\sqrt{{\varepsilon }_{r1}}}\mathrm{tan}((\omega \sqrt{{\varepsilon }_{r1}}){t}_{d}/c)}{\frac{{Z}_{0}}{\sqrt{{\varepsilon }_{r1}}}+j{Z}_{2}\mathrm{tan}((\omega \sqrt{{\varepsilon }_{r1}}){t}_{d}/c)}$$

where, $${\beta }_{1}=(\omega \sqrt{{\varepsilon }_{r1}})/c,{\varepsilon }_{r1} \mathrm{\hspace{0.1cm} and\hspace{0.1cm}} {t}_{d}$$ is the phase constant, complex dielectric constant and thickness of FR-4 dielectric substrate, respectively. From Fig. 1c input impedance of the proposed absorbing structure ($${Z}_{in})$$ can be written as

6 $${Z}_{in}={Z}_{MA}|| {Z}_{1} =\frac{{Z}_{MA} {Z}_{1} }{{Z}_{MA}+ {Z}_{1}}$$

Therefore, the reflection coefficient of the absorbing structure ($\Gamma \left(\omega \right))$ can be written as

7 $$\Gamma \left(\omega \right)= \frac{{Z}_{in}-{Z}_{0}}{{Z}_{in}+{Z}_{0}}$$

When the admittance of the top metallic layer (${Y}_{MA}$) have equal and opposite (in sign) magnitude with respect to admittance of the substrate layer (${Y}_{1}$) just below top metallic layer, then this parallel circuit will resonate. This will result into purely real value of total input impedance ${Z}_{in}$. Now when this real value of input impedance ${Z}_{in}$ matches free space impedance ${Z}_{0}$, the reflection coefficient becomes zero and we get unity absorptivity.

To investigate the performance of the proposed design of metasurface absorber, we have used CST MICROWAVE STUDIO. Numerical simulation has been done in frequency domain solver. Also, unit cell boundary condition has been used in x- and y-direction to imitate infinite periodic array and open boundary conditions along z-direction.

**Figure 2:**
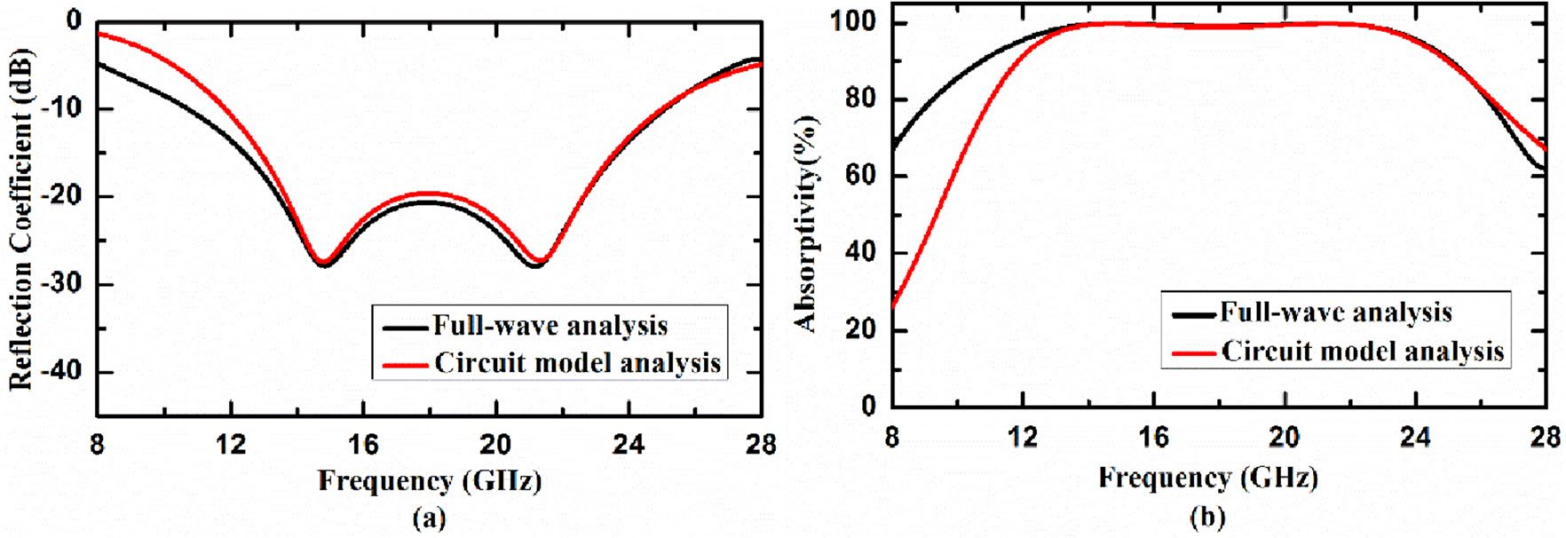
(**a**) Reflection coefficient and (**b**) absorptivity of the proposed metasurface absorber calculated using equivalent circuit model analysis and full-wave simulation using CST MICROWAVE STUDIO.

Further we have used CST DESIGN STUDIO to verify equivalent circuit modeling of the proposed absorber. Parameters of circuit model have been extracted by fitting amplitude and phase of reflection coefficient with full wave simulated results. Extracted parameters are *R*_*MA1*_ = 319.507Ω, *L*_*MA1*_ = 3.79 nH, *C*_*MA1*_ = 0.0213075 pF, *R*_*MA2*_ = 83.9688Ω, *L*_*MA2*_ = 3.412nH, and *C*_*MA2*_ = 0.007822 pF. Reflection coefficient and absorptivity of the proposed metasurface absorber calculated using equivalent circuit model analysis and full-wave simulation using CST MICROWAVE STUDIO have been shown in Fig. 2a,b, respectively. It is observed from figure that results calculated from equivalent circuit modeling matches very well with full wave simulated results. Numerical simulation shows 20 dB reflection reduction (which corresponds to 99% absorptivity) from 13.42 to 22.66 GHz under normal incidence.

### Absorption characteristics under different polarization and incidence angle

Absorption capabilities of the proposed metasurface absorber has been studied under variations of the polarization angle and angle of incidence to test its reliability and applicability as a standard device.

**Figure 3:**
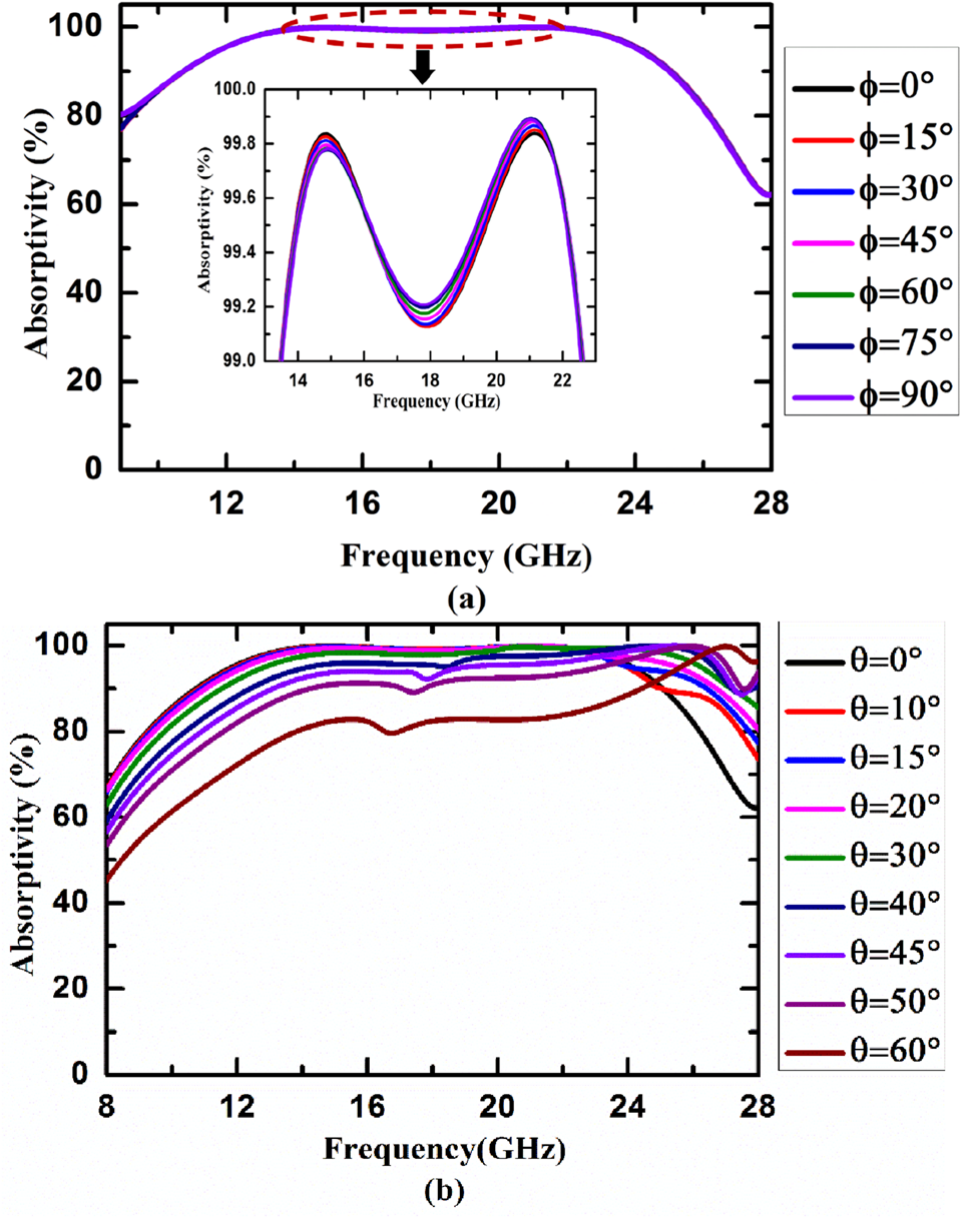
Absorption characteristic of the proposed metasurface absorber under different (**a**) polarization angle (ϕ) and (**b**) angle of incidence (θ) of TE-polarized wave.

A. *Variation of the polarization angle*
  In order to use the proposed metasurface absorber as a standard absorbing device in radar stealth applications, its absorption characteristics must be independent of the polarization of incoming EM wave. To satisfy this requirement, the unit cell of the proposed metasurface absorber has been designed to exhibit four-fold rotation symmetry around the propagation direction of incoming EM wave. The polarization independent characteristic of the proposed metasurface absorber has been further validated by studying its absorption response under different angles of polarization of incoming wave as depicted in Fig. 3a.
B. *Variation of the incident angle*
  The performance of the proposed metasurface absorber under different oblique incidences is also studied. The graphical representation of numerically simulated absorptivity of the proposed absorber by varying incidence angle of TE-polarized wave from 0° to 60° is shown in Fig. 3b. It has been found that proposed absorber maintains more than 90% absorptivity up to 40° angle of incidence in the given frequency regime (13.42 to 22.66 GHz).

### Explanation of absorption mechanism

**Figure 4:**
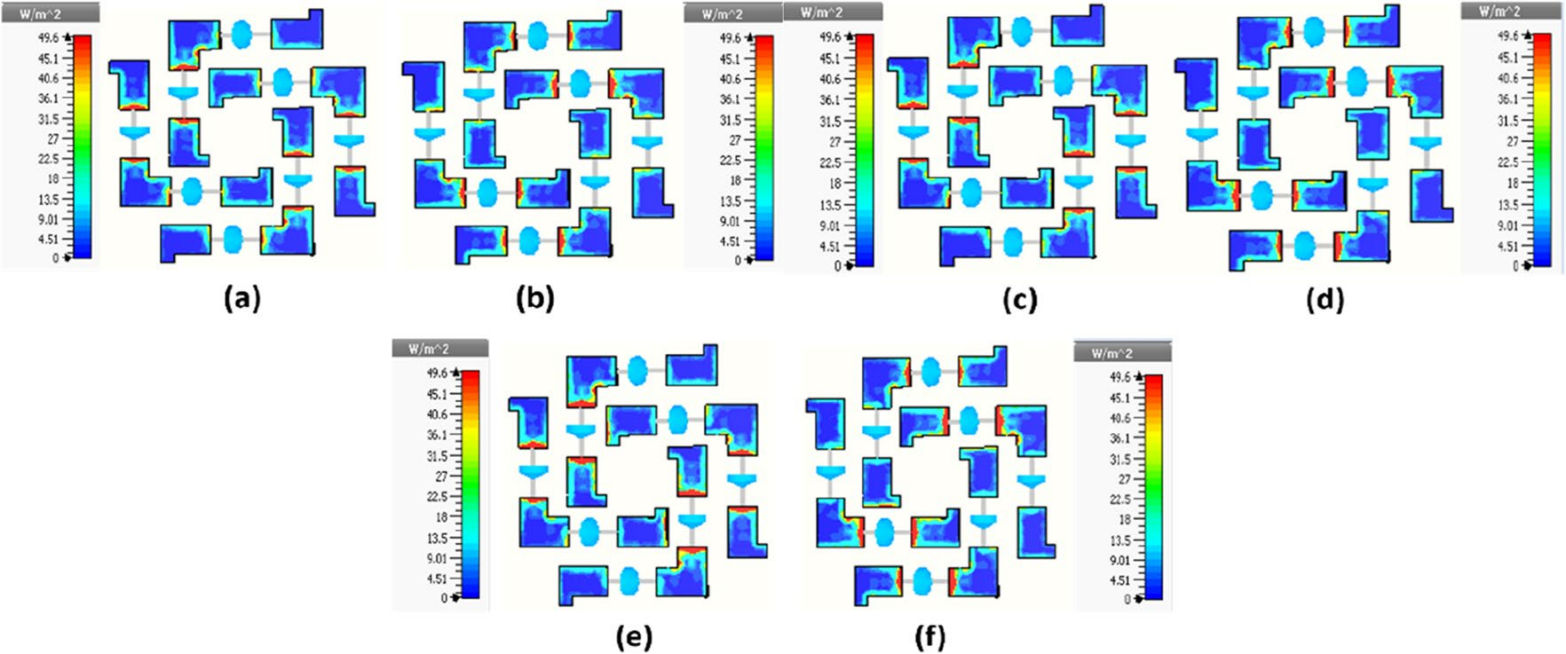
Power loss density distribution at (**a**) 14.84 GHz, (**c**) 17.99 GHz, (**e**) 21.14 GHz for TE- polarized incidence, and power loss density distribution at (**b**) 14.84 GHz, (**d**) 17.99 GHz, (**f**) 21.14 GHz for TM polarized incidence.

To get the insight of physics behind the absorption mechanism, we studied the power loss density distribution at 14.84 GHz, 17.99 GHz and 21.14 GHz (two reflection minima and mid frequency), illustrated in Fig. 4.

The power loss density distribution at 14.84 GHz, 17.99 GHz, and 21.14 GHz for TE-polarized incidence are shown in Fig. 4a,c,e, respectively, and power loss density distribution at 14.84 GHz, 17.99 GHz, 21.14 GHz for TM-polarized incidence are shown in Fig. 4b,d,f, respectively. It is observed in figure that for TE-polarized incidence, power loss density is maximum around the resistors that is connected to vertical arms of metallic patch and for TM-polarized incidence, the power loss density is maximum around the resistors that is connected to horizontal arms of metallic patch. From these observations it is indicated that most of the energy dissipation is due to ohmic losses of the lumped resistors mounted on metallic patches.

### RCS reduction analysis of the proposed metasurface absorber

In radar stealth application, the RCS of the potential targets like aircraft, must be low to disable its detection from enemy’s radar and thus enables its successful surveillance. Since the major contributors towards the RCS of targets are planar, cylindrically bent and 90° dihedral surfaces, therefore it is required to study the RCS reduction capabilities of the proposed metasurface absorber when applied on these surfaces.

In this section, we have studied RCS reduction capabilities of the proposed metasurface absorber on above mentioned surfaces. RCS of the proposed metasurface absorber is compared with same size and shape perfect electric conductor (PEC).

The RCS, σ, of a target is defined as^1^:

8 $$\upsigma = 4\uppi {\mathrm{lim}}_{R\to \infty }{R}^{2}\frac{{|{E}_{s}|}^{2}}{{|{E}_{i}|}^{2}}$$

where, R is the distance between the target and the detection radar, *E*_*i*_ is the electric field intensity of the incident wave, and *E*_*s*_ is the electric field intensity of the scattered waves from the target. RCS of the metasurface absorber can be calculated using electric field monitoring system in CST MICROWAVE STUDIO with the help of Eq. (8).

### Case I. Metasurface absorber for planar surface

**Figure 5:**
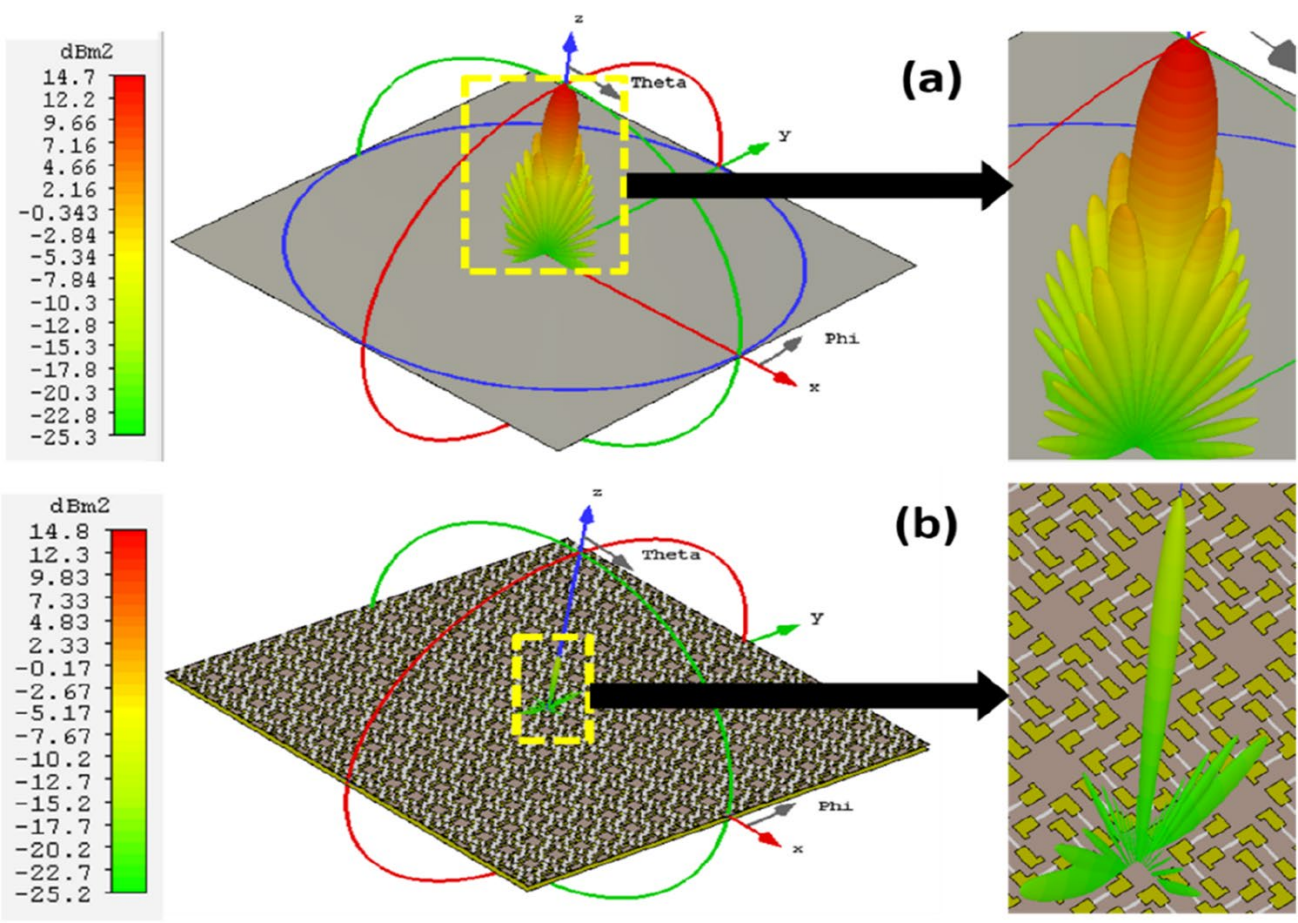
3D RCS patterns of (**a**) planar PEC and (**b**) planar MA at 14.84 GHz under normal incidence.

**Figure 6:**
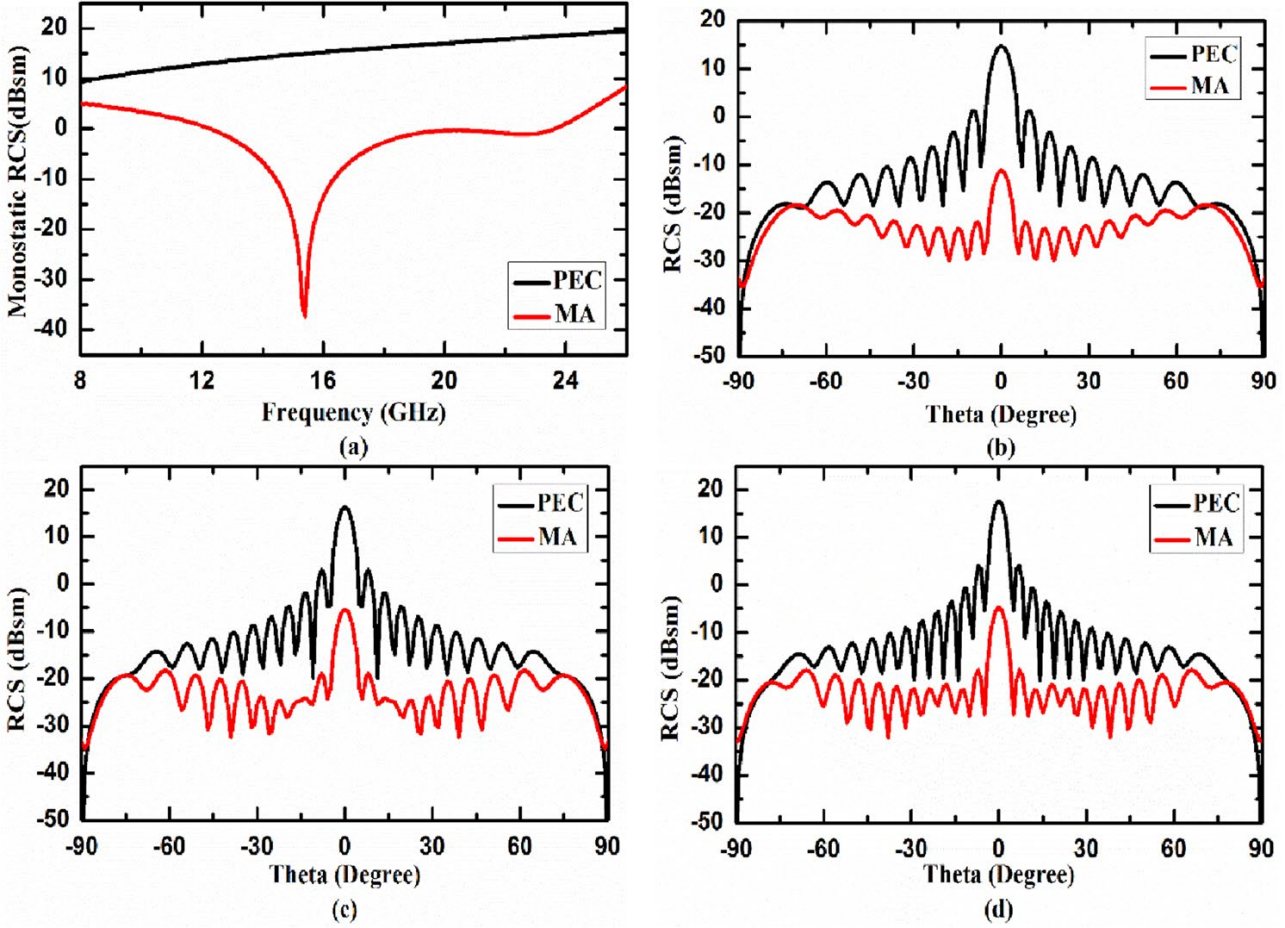
(**a**) Simulated monostatic RCS of the proposed planar MA and PEC under normal incidence, bistatic RCS of PEC and MA at (**b**) 14.84 GHz, (**c**) 17.99 GHz, (**d**) 21.14 GHz under normal incidence.

In this section, we have used 16 × 16 unit cells of the proposed metasurface absorber (MA) for analyzing its RCS reduction capabilities for both monostatic as well as bistatic radar system. PEC plate with same area as of metasurface absorber has been used for reference value. 3D RCS patterns of planar PEC and planar MA at 14.84 GHz under normal incidence are shown in Fig. 5a,b, respectively. To find the RCS reduction capability of MA under broad frequency spectrum we calculated monostatic RCS of both MA and PEC, where both transmitter and receiver antennas are collocated. In Fig. 6a, the monostatic RCS of the planar PEC plate (shown by black curve) and planar MA (shown by red curve) as a function of frequency under normal incidence are plotted. It is evident from the figure that proposed absorber achieved nearly 20 dB RCS reduction in the given frequency band (13.42–22.66 GHz) as compared to PEC plate under normal incidence. To study the performance of planar MA under different observation (receiving antenna) angles, we have calculated bistatic RCS of planar MA and planar PEC under normal incidence. In this case, transmitting antenna is kept at 0° with respect to the normal to the surface of MA and receiving antenna is scanned from 0° to 90° with respect to the normal. As we can see in Fig. 2a, we have resonances at 14.84 GHz and 21.14 GHz with centre frequency at 17.99 GHz. Therefore, we have calculated the bistatic RCS of MA and PEC at these frequencies. In Fig. 6b–d, the bistatic RCS of the planar PEC plate (shown by black curve) and planar MA (shown by red curve) as function of receiving angle are plotted at 14.84 GHz, 17.99 GHz and 21.14 GHz, respectively. As we can see from Fig. 6b–d the proposed MA significantly reduces the RCS at all the given frequencies.

### Case II. Metasurface absorber for cylindrically bent surface

**Figure 7:**
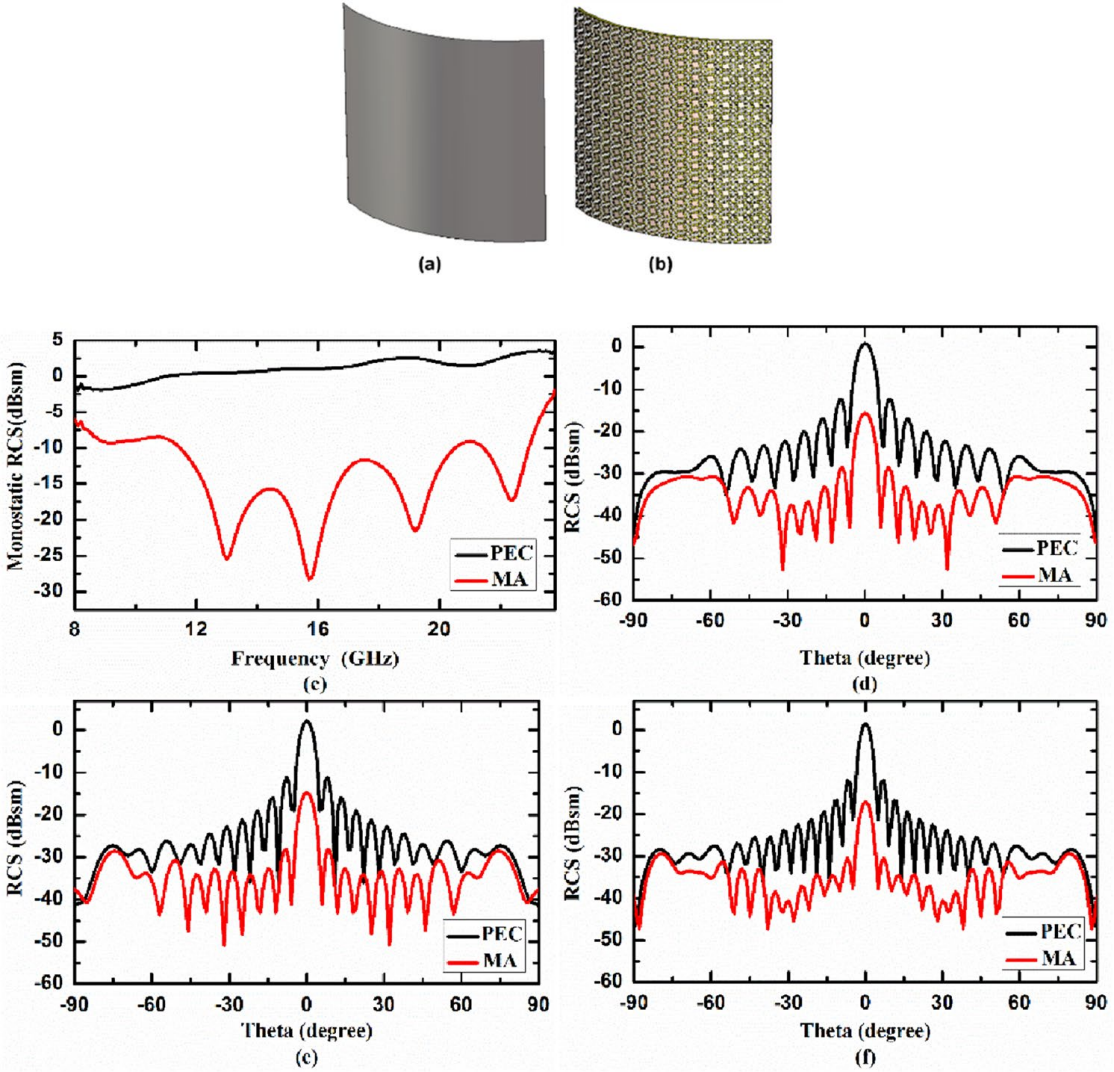
3D view of cylindrically bent (**a**) PEC and (**b**) MA with radius of curvature 130 mm, (**c**) monostatic RCS, and bistatic RCS at (**d**) 14.84 GHz, (**e**) 17.99 GHz, (**f**) 21.14 GHz under normal incidence.

**Figure 8:**
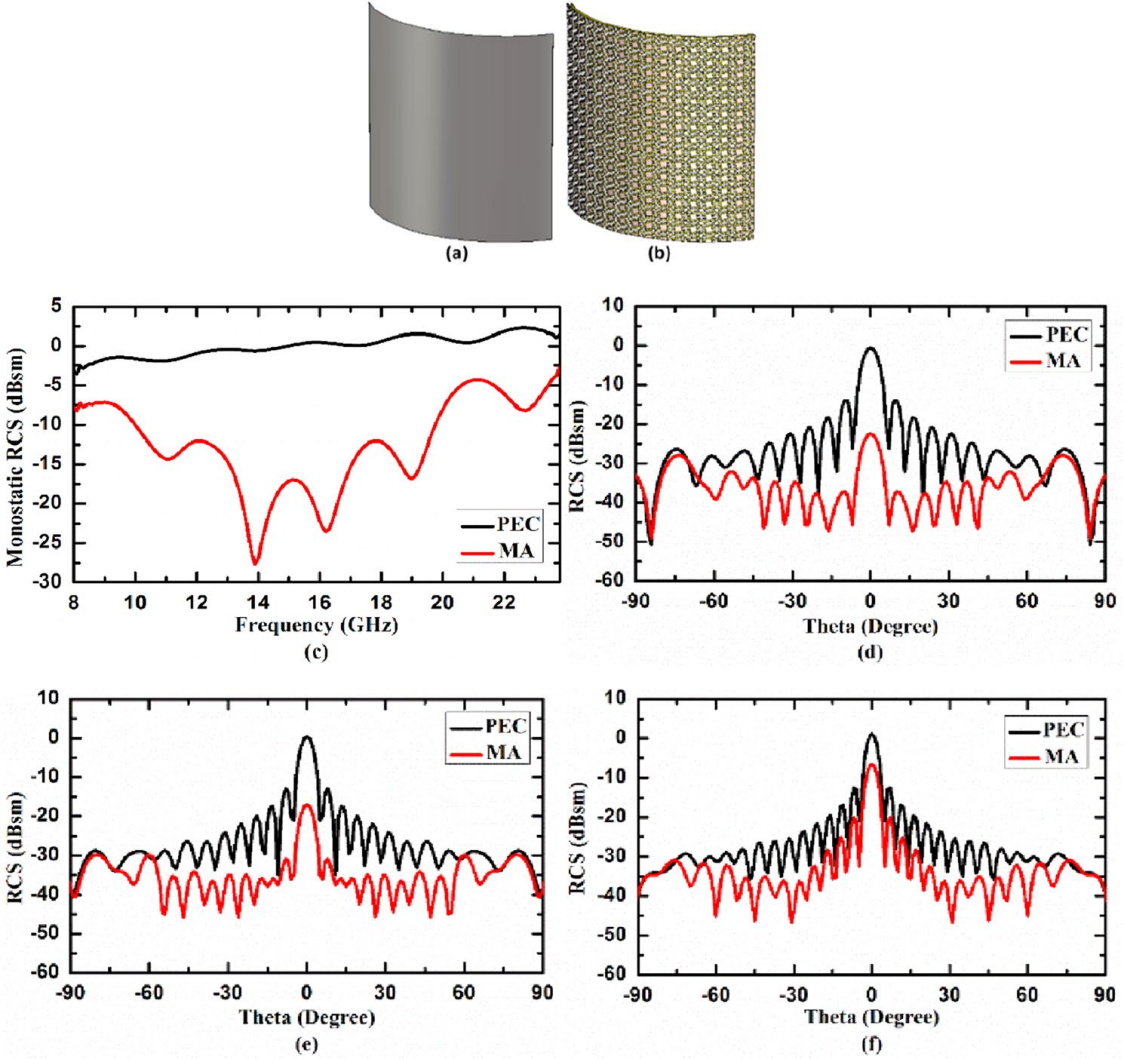
3D view of cylindrically bent (**a**) PEC and (**b**) MA with radius of curvature 100 mm, (**c**) monostatic RCS, and bistatic RCS at (**d**) 14.84 GHz, (**e**) 17.99 GHz, (**f**) 21.14 GHz under normal incidence.

**Figure 9:**
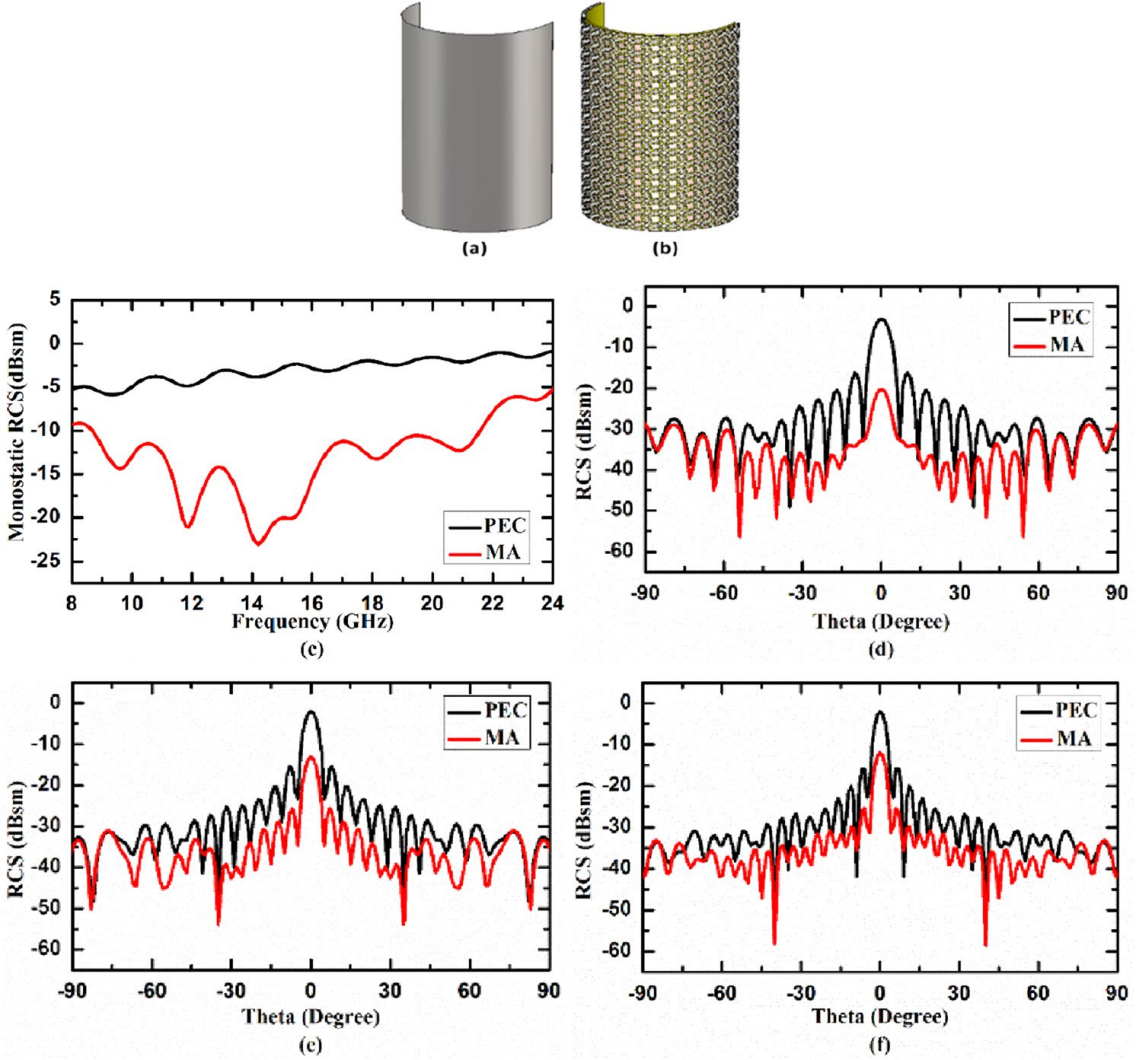
3D view of cylindrically bent (**a**) PEC and (**b**) MA with radius of curvature 50 mm, (**c**) monostatic RCS, and bistatic RCS at (**d**) 14.84 GHz, (**e**) 17.99 GHz, (**f**) 21.14 GHz under normal incidence.

In second case, we studied the RCS reduction capability of the proposed MA on cylindrically bent surface for three different radius of curvature (r) i.e. r = 130 mm, 100 mm, 50 mm, consisting 16 × 16 unit cells. We first calculated RCS reduction capability of cylindrically bent MA with radius of curvature, r = 130 mm. To have a baseline reference, the RCS of cylindrically bent MA is compared with the same size perfect electric conductor (PEC) which is also bent in cylindrical surface with radius of curvature (r) 130 mm. 3D view of cylindrically bent PEC and MA with radius of curvature 130 mm are shown in Fig. 7a,b, respectively. In Fig. 7c, the monostatic RCS of the cylindrically bent PEC and MA with radius of curvature 130 mm as a function of frequency under normal incidence are plotted. Figure 7d–f shows the bistatic RCS of cylindrically bent PEC and MA at 14.84 GHz, 17.99 GHz and 21.14 GHz, respectively, as function of receiving angle under normal incidence. From the figure it is evident that the RCS of the proposed cylindrically bent MA (shown by red curve) is significantly lower than RCS of PEC (shown by black curve) for 130 mm radius of curvature. We further decreased the radius of curvature to 100 mm and then to 50 mm, to study the effect of bending on the performance of metasurface absorber. Figure 8a,b depicts the 3D view of cylindrically bent PEC and MA, respectively, with radius of curvature 100 mm, while Fig. 8c depicts the monostatic RCS of these surfaces under normal incidence. Figure 8d–f shows the bistatic RCS of cylindrically bent PEC and MA at 14.84 GHz, 17.99 GHz and 21.14 GHz, respectively, under normal incidence. Similarly, Fig. 9a,b shows 3D view of cylindrically bent PEC and MA, respectively, with radius of curvature r = 50 mm and Fig. 9c shows the monostatic RCS of these surfaces under normal incidence. The bistatic RCS of these surfaces at 14.84 GHz, 17.99 GHz and 21.14 GHz under normal incidence are shown in Fig. 9d–f, respectively. From the figures it is observed that under 100 mm and 50 mm radius of curvature also the RCS of the designed cylindrically bent MA (shown by red curve) is significantly lower than RCS of PEC (shown by black curve). From these case studies it is concluded that the designed MA can significantly reduce RCS in cylindrical surfaces as well for different radius of curvature. From figure it is also observed that as the radius of curvature is reduced from 130 to 100 mm and then to 50 mm, the performance of MA on cylindrically bent surfaces in terms of RCS reduction value deteriorates. In other words, we can say that the cylindrically bent MA with larger radius of curvature performs better as compared to smaller radius of curvature.

### Case III: Metasurface absorber on 90° dihedral surface

**Figure 10:**
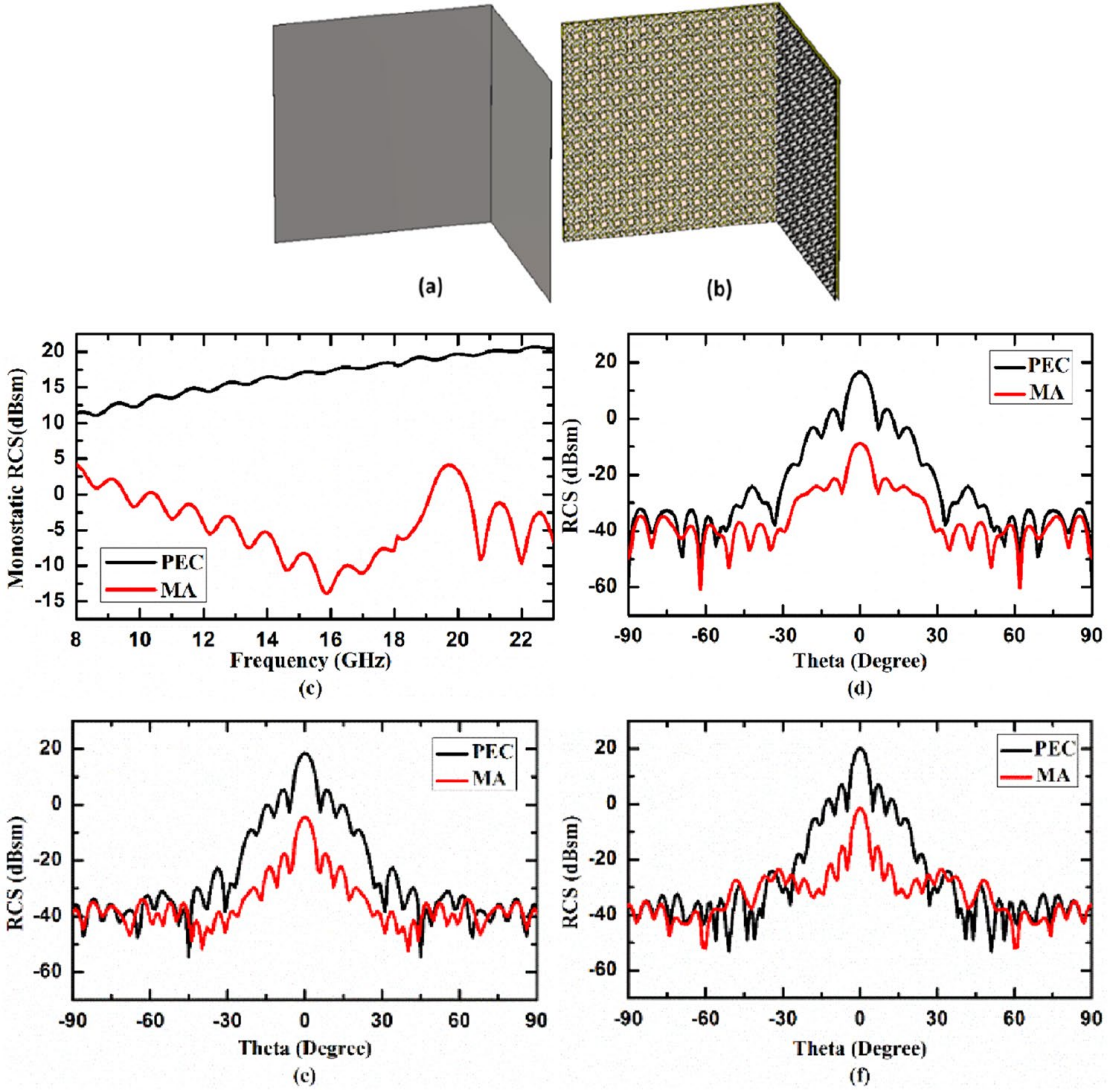
3D view of 90° dihedral (**a**) PEC and (**b**) MA, (**c**) monostatic RCS, and bistatic RCS at (**d**) 14.84 GHz, (**e**) 17.99 GHz, (**f**) 21.14 GHz under normal incidence.

In third case study, RCS reduction capability of the proposed MA on 90° dihedral surface with 16 × 16 unit cells on both side is studied and compared it with same size 90° dihedral PEC surface. Figure 10a, b illustrates 3D view of 90° dihedral PEC and MA surface, respectively. In Fig. 10c, the monostatic RCS of the 90° dihedral PEC surface (shown by black curve) and 90° dihedral MA surface (shown by red curve) as a function of frequency under normal incidence are plotted. The bistatic RCS of 90° dihedral PEC and MA surface at 14.84 GHz, 17.99 GHz and 21.14 GHz as function of receiving angle under normal incidence are shown in Fig. 10d–f, respectively. From figure it is observed that RCS of MA on 90° dihedral surface is significantly reduced as compared to PEC. Therefore, from these case studies where proposed MA is applied on different types of geometries and its RCS is compared with PEC (with same size and geometry as MA), it can be inferred that the proposed metasurface absorber is capable of reducing RCS significantly and thus can be used in radar stealth application.

## Experimental results

### Reflection reduction characteristics of developed planar metasurface

**Figure 11:**
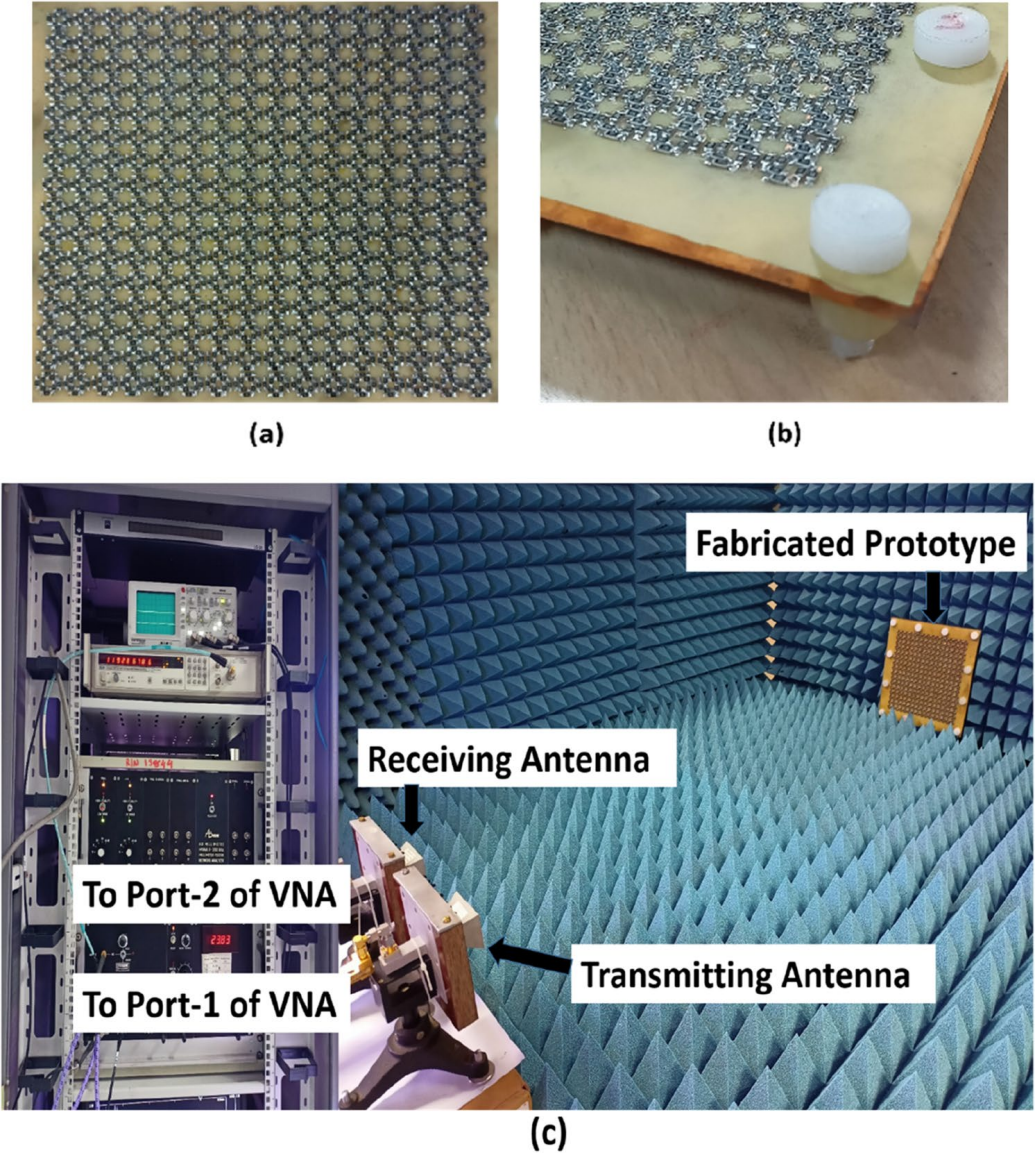
(**a**) Top view and (**b**) 3D view of fabricated prototype, (**c**) experimental setup.

To validate the numerically simulated results, a sample with 16 × 16 unit cell of the proposed design of metasurface absorber has been fabricated using PCB technology. The top metallic layer has been etched to form metallic patches on top of a FR-4 dielectric substrate having thickness of 0.2 mm. After this process, the chip resistors with a value of 150 Ω (SMD Resistor—1206 Package, a type of surface mount and a standard thick film chip resistor which works well in the given frequency band) are soldered on top of metallic patches using surface mounting technology. We have used Teflon screws with spacer thickness of 2.6 mm to hold and separate this top resistively loaded metallic layer on FR-4 substrate by another metallic ground plate. Figure 11a,b shows the top view and 3D view of the fabricated prototype, respectively. Figure 11c shows the experimental setup that consists of two horn antenna (transmitter and receiver) connected to AB*mm* vector network analyzer (VNA) and device under test (DUT)/fabricated prototype. Using the free-space measurement technique, two linearly polarized standard horn antenna are connected to port-1 (transmission port) and port 2 (receiver/detection port) of the VNA. The DUT has been placed at a distance where far-field condition and anechoic chamber standards are satisfied.

**Figure 12:**
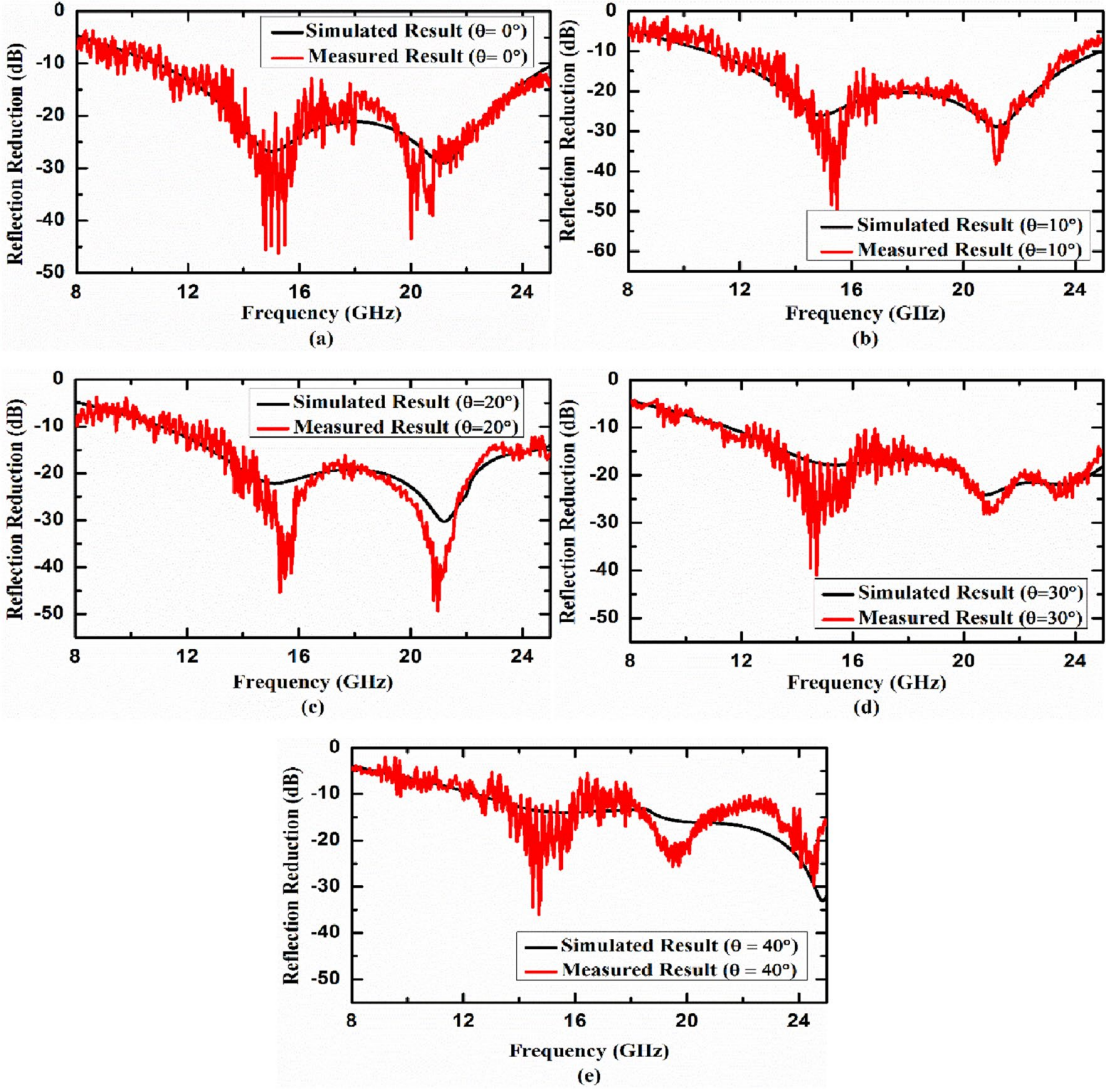
Simulated and measured reflection reduction characteristic of the developed planar metasurface absorber under (**a**) normal incidence, (**b**) 10°, (**c**) 20°, (**d**) 30° and (**e**) 40° angle of incidence (specular measurement).

Before the actual measurements to calculate reflection reduction, the measurement setup through VNA is calibrated to include the transmission path losses, diffraction losses etc. This calibration is done by placing a copper plate of same size as DUT and at the same far field location. After calibration, the copper plate is replaced with DUT and reflection reduction measurements are calculated thereafter. The simulated and measured reflection reduction of the developed planar metasurface absorber under normal incidence are shown in Fig. 12a. We achieved the same 20 dB reflection reduction bandwidth as we get in numerical simulations. The fabricated prototype has been also experimentally tested for incidence angle sensitivity. The simulated and measured reflection reduction characteristics of developed planar metasurface absorber as a function of frequency (specular measurement) for 10°, 20°, 30° and 40° angle of incidence are shown in Fig. 12b–e, respectively. It is evident from the figures that the measured reflection reduction under normal incidence and oblique incidence has a very good agreement with simulated results and the developed device exhibits wide incidence angle stability. Minor deviations from the simulated results can be attributed to fabrication tolerances, manual soldering of SMD chip resistors and measurement noise which are not included in simulations. The measured data shown here is raw and can be smoothened out using smoothening technique. The noise that is seen in the measured results is a combination of many factors like (1) exact alignment of the receiving antenna with respect to testing sample/DUT, (2) reflections/scattering from near field regions, (3) connector and cable losses, (4) instrumentation noise etc.

### Reflection reduction characteristics of developed conformal metasurfaces

In radar stealth application, the target have planar as well as non-planar surfaces over which metasurface absorber needs to be applied to reduce its RCS. Thus, it is important that the RCS reduction characteristics of developed metasurface absorber should satisfy for both planar as well as conformal surfaces. To validate this requirement, the developed planar metasurface has now been reconfigured in cylindrically bent and 90° dihedral metasurfaces.

**Figure 13:**
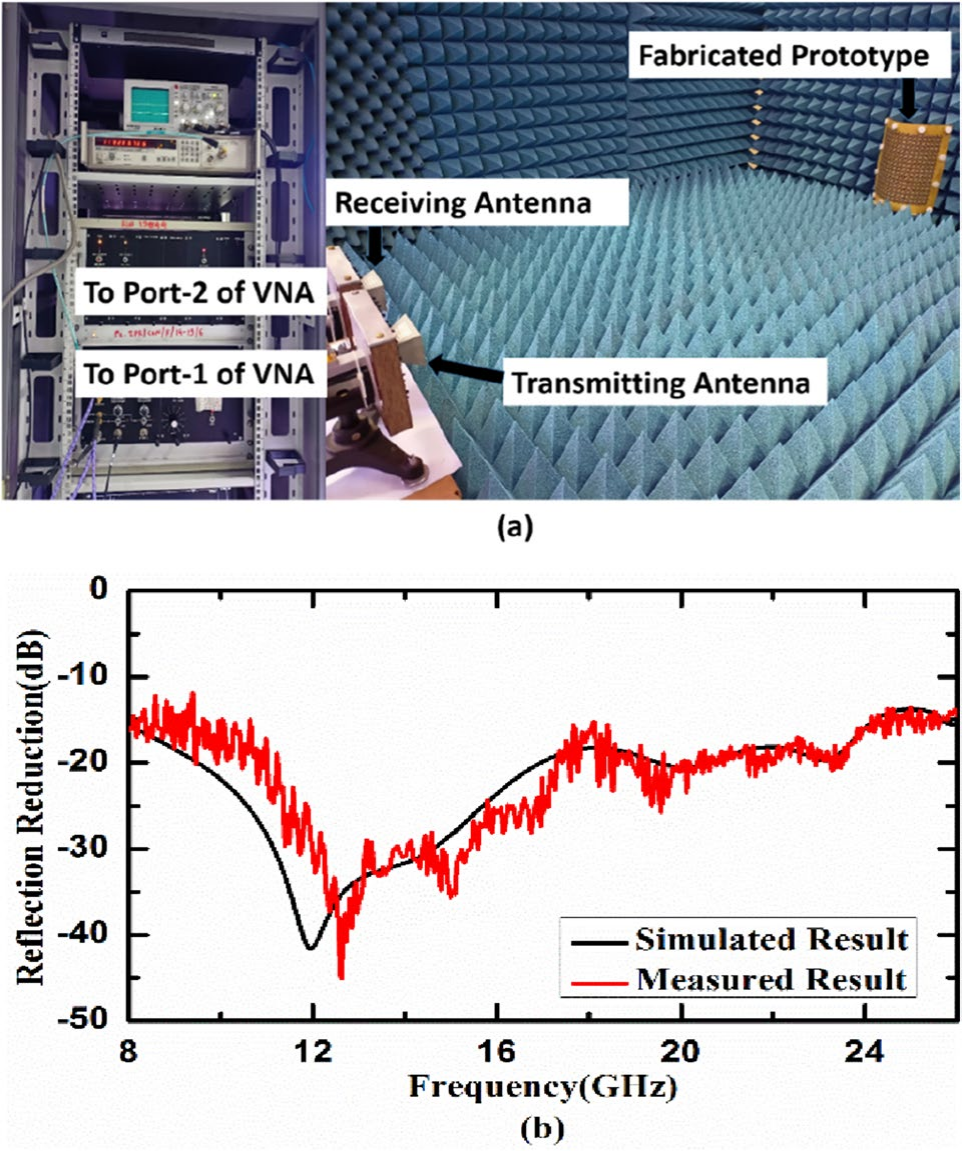
(**a**) Experimental setup for cylindrically bent surface with radius of curvature 130 mm and (**b**) simulated and measured reflection reduction of the developed cylindrically bent metasurface under normal incidence.

**Figure 14:**
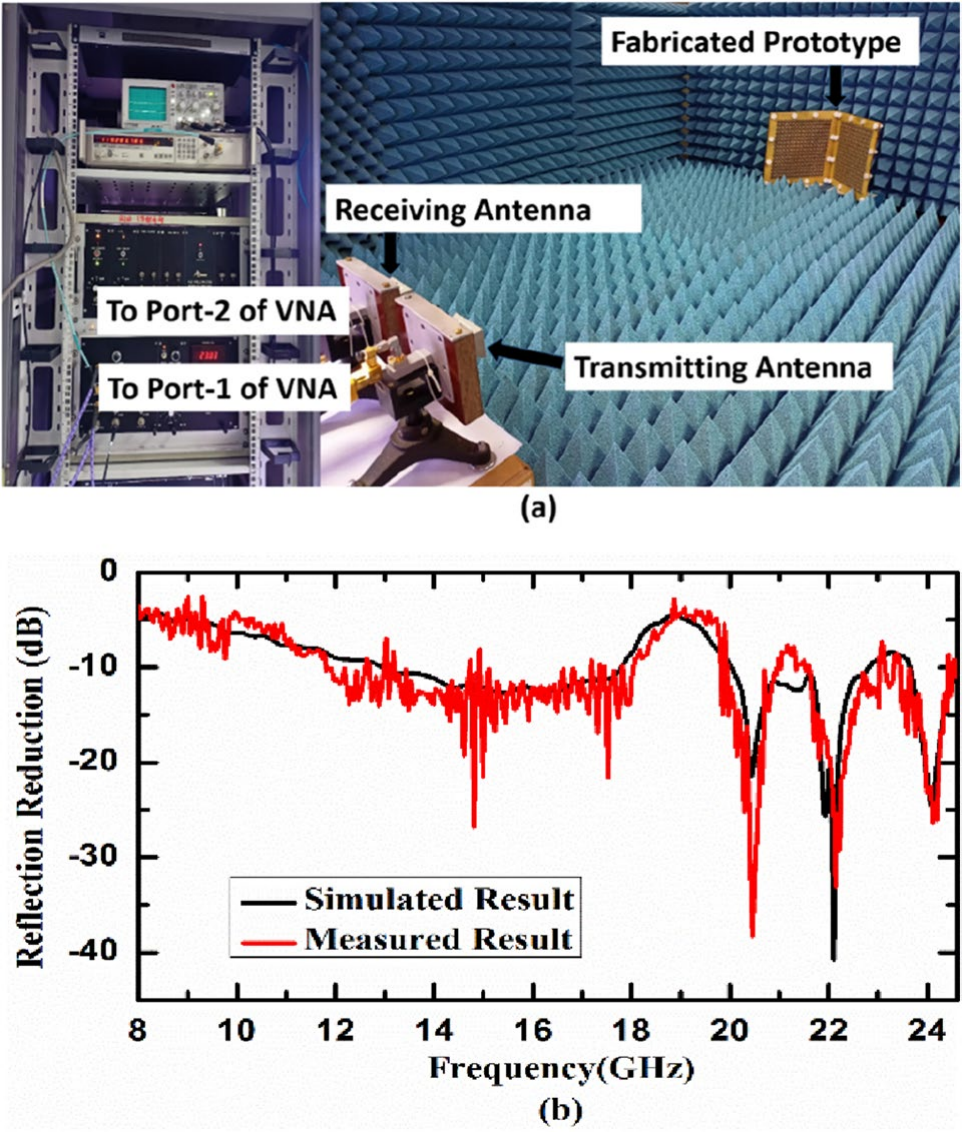
(**a**) Experimental setup for 90° dihedral metasurface and (**b**) simulated and measured reflection reduction of the developed 90° dihedral metasurface under normal incidence.

The developed cylindrically bent metasurface with 130 mm radius of curvature and the experimental setup for its characterization are depicted in Fig. 13a. Figure 13b shows the simulated and measured reflection reduction of cylindrically bent metasurface with 130 mm radius of curvature under normal incidence. It is observed from the figure that numerically simulated result matches well with measured result and we obtained the same 20 dB reflection reduction bandwidth as we get in numerical simulations. Similarly, the developed 90° dihedral metasurface and the experimental setup for its characterization are depicted in Fig. 14a. Figure 14b shows the simulated and measured reflection reduction for 90° dihedral metasurface under normal incidence. It is evident from the graph that numerically simulated result matches well with measured result. Minor deviations from the simulated results can be attributed to fabrication tolerances, manual soldering of SMD chip resistors and measurement noise which are not included in simulations. The measured data shown here is raw and can be smoothened out using smoothening technique. The noise that is seen in the measured results is a combination of many factors like (1) exact alignment of the receiving antenna with respect to testing sample/DUT, (2) reflections/scattering from near field regions, (3) connector and cable losses, (4) instrumentation noise etc.

In Table 1, we compared the performance of the developed device with all the previously reported work. It is observed from the table that it provides better reflection reduction value as compared to^12–18,21,22^. The developed device provides wider 20 dB reflection reduction bandwidth (51.21%) compared to^20^. Although Refs.^19,23^. proposes 20 dB reflection reduction but experimental validation is provided only for 10 dB reflection reduction.

**Table 1:** Performance of developed device with previous reported work.

References	Type	Reflection reduction bandwidth	RCS reduction	Polarization insensitivity
^12^	Lumped element	45.6% (10 dB)	Not reported	Yes
^13^	Lumped element	51.16% (10 dB)	Not reported	Yes
^14^	Lumped element	60.3% (10 dB)	Not reported	Yes
^15^	Lumped element	70.07% (10 dB)	Not reported	Yes
^16^	Lumped element	73.6% (10 dB)	Not reported	Yes
^17^	Lumped element	86% (10 dB)	Not reported	No
^18^	Lumped element	117% (10 dB)	Not reported	Yes
^19^	Lumped element	126.8%(10 dB) and 83.4% (20 dB)^a^	Not reported	Yes
^20^	Lumped element	40% (20 dB)	Not reported	No
^21^	Resistive ink	120%(10 dB)	Reported for planar surface	Yes
^22^	Resistive ink	96.29% (15 dB)	Not reported	Yes
^23^	Resistive ink	68.69%(20 dB)^a^	Not reported	Yes
This work	Lumped element	51.21% (20 dB)	Planar, cylindrically bent and 90° dihedral surface	Yes

^a^Measurement reported only for 10 dB reflection reduction.

## Discussion and conclusion

We have successfully designed, developed and characterized a broadband polarization-insensitive metasurface absorber. The designed structure is composed of metallic patches loaded with lumped resistor on top of FR-4 dielectric substrate separated by metallic ground plate using an air spacer. Numerical simulation shows that the proposed metasurface absorber exhibits 20 dB reflection reduction with 51.21% fractional bandwidth from 13.42 to 22.66 GHz under normal incidence. Equivalent circuit modeling of the proposed absorbing structure is studied to understand and validate numerical findings which are in good agreement. Due to fourfold rotation symmetry, the absorption characteristics of the proposed absorber is polarization insensitive. Power loss density distribution of the proposed absorber have been studied to explain the physics behind absorption mechanism. Further extending our studies, the RCS reduction capability of the proposed absorber on planar, cylindrically bent and 90*°* dihedral surface have been extensively analyzed and it has been observed that the proposed metasurface absorber significantly reduces RCS of the target for all geometries. The designed metasurface absorber is fabricated in planar, cylindrically bent and 90*°* dihedral surface geometries and characterized using AB*mm* VNA and free space measurement method. Measured reflection reduction characteristics of the developed metasurface absorber are in good agreement with numerical-analytical findings. Minor deviations from the simulated results can be attributed to fabrication tolerances, manual soldering of SMD chip resistors and measurement noise which are not included in simulations. The measured data shown here is raw and can be smoothened out using smoothening technique. The noise that is seen in the measured results is a combination of many factors like (1) exact alignment of the receiving antenna with respect to testing sample/DUT, (2) reflections/scattering from near field regions, (3) connector and cable losses, (4) instrumentation noise etc. However, the error is minor within ± 2–5% that can be removed. From these studies, it is inferred that the proposed metasurface absorber can be efficiently used in radar stealth technology as a standard absorbing device.

## Methods

### Fabrication method

A sample with 16 × 16 unit cell of the proposed design of metasurface absorber has been fabricated using printed circuit board (PCB) technology. The top copper layer of single sided FR-4 dielectric substrate with thickness of 0.2 mm has been etched to form metallic patches. Once PCB is ready, the chip resistors with a value of 150 Ω (SMD Resistor—1206 Package, a type of surface mount and a standard thick film chip resistor) are soldered at required position on top of metallic patches using surface mounting technology. Now this top resistively loaded metallic layer on FR-4 substrate is separated from ground metallic plate (copper plate) using teflon screws with spacer thickness 2.6 mm.

### Measurement method

The experimental setup for characterization of developed metasurface absorber consists of two horn antenna (transmitter and receiver) connected to AB*mm* VNA and DUT. Using the free-space measurement method, two linearly polarized standard horn antenna are connected to port-1 (transmission port) and port 2 (receiver/detection port) of the VNA. The DUT has been placed at a distance where far-field condition and anechoic chamber standards are satisfied. Before the actual measurements to calculate reflection reduction, the measurement setup through VNA is calibrated to include the transmission path losses, diffraction losses etc. This calibration is done by placing a copper plate of same size as DUT and at the same far field location. After calibration, the copper plate is replaced with DUT and reflection reduction measurements are calculated thereafter.

## Data Availability

The data supporting the findings of this study available from the corresponding author on reasonable request.
